# Light-Scattering Simulations from Spherical Bimetallic Core–Shell Nanoparticles

**DOI:** 10.3390/mi12040359

**Published:** 2021-03-26

**Authors:** Francesco Ruffino

**Affiliations:** Dipartimento di Fisica e Astronomia “Ettore Majorana”, Università di Catania, CNR-IMM, via S. Sofia 64, 95123 Catania, Italy; francesco.ruffino@ct.infn.it

**Keywords:** bimetallic nanoparticles, core–shell nanoparticles, light-scattering, Mie theory, scattering efficiency

## Abstract

Bimetallic nanoparticles show novel electronic, optical, catalytic or photocatalytic properties different from those of monometallic nanoparticles and arising from the combination of the properties related to the presence of two individual metals but also from the synergy between the two metals. In this regard, bimetallic nanoparticles find applications in several technological areas ranging from energy production and storage to sensing. Often, these applications are based on optical properties of the bimetallic nanoparticles, for example, in plasmonic solar cells or in surface-enhanced Raman spectroscopy-based sensors. Hence, in these applications, the specific interaction between the bimetallic nanoparticles and the electromagnetic radiation plays the dominant role: properties as localized surface plasmon resonances and light-scattering efficiency are determined by the structure and shape of the bimetallic nanoparticles. In particular, for example, concerning core-shell bimetallic nanoparticles, the optical properties are strongly affected by the core/shell sizes ratio. On the basis of these considerations, in the present work, the Mie theory is used to analyze the light-scattering properties of bimetallic core–shell spherical nanoparticles (Au/Ag, AuPd, AuPt, CuAg, PdPt). By changing the core and shell sizes, calculations of the intensity of scattered light from these nanoparticles are reported in polar diagrams, and a comparison between the resulting scattering efficiencies is carried out so as to set a general framework useful to design light-scattering-based devices for desired applications.

## 1. Introduction

Metallic nanostructures have acquired a fundamental role in the modern nanotechnology revolution [1,2,3,4,5,6,7,8,9]. They are now routinely exploited in functional, innovative device finding applications in several technological areas ranging from energy production and storage, sensing, electronics, health, etc. [1,2,3,4]. In particular, the peculiar optical properties of metallic nanoparticles (NPs) are effectively exploited in several devices as energy, photonics and sensing ones [1,2,3,4,10,11,12,13,14,15,16,17]. For example, metallic NPs show strong optical resonances: under excitation by the electromagnetic radiation, metallic NPs can exhibit localized surface plasmon resonance (LSPR) due to the collective oscillations of conduction electrons [1,2,3,4]. The surface plasmon of a metal is a collective excitation of electrons in the conduction band, and they dominate the electromagnetic responses of the metallic system with sizes on the order of the plasmon resonance wavelength. This phenomenon occurs when the electromagnetic field interacts with conduction band electrons and induces the coherent oscillation of electrons. Consequently, a strong absorption band appears in some regions of the electromagnetic spectrum depending on the size and shape of the metallic structure. Hence, the peculiar consequences of the resonant excitation of LSPR rely upon selective photon absorption and enhancement of local electromagnetic fields near the NPs by orders of magnitude. This approach allows, for example, to control wavelengths to which the LSPR occurs through the visible to the near-infrared region simply by tuning the NPS shape and size. Such a characteristic makes metal NPs very important plasmonic solar cells [1,2,3,4,15,16,17,18,19] and in chemical and biological sensing, exploiting the surface-enhanced Raman spectroscopy effect [1,2,3,4,14,15,16,17]. In addition to their LSPR absorption, light-scattering is another optical characteristic of metallic NPs that is of great interest [1,2,3,4,18,19,20,21]. In fact, metal NPs under electromagnetic radiation of a specific wavelength (λ_0_) that matches the plasmon absorption maxima (λ_p_), in addition to absorb light, can also scatter light outside of their physical cross-sections. The light-scattering by metallic NPs finds some interesting applications as in biological and molecular recognition [22,23,24], in energy production by photovoltaic devices to increase the light trapping in thin-film silicon and organic solar cells for achieving a higher photocurrent [10,11,12,13]. Generally, by controllably changing the composition, shape and size of metallic NPs, it is possible to design nanostructures absorbing light in the desired wavelength range. As the shape or size of the nanoparticles changes, the surface geometry changes causing a shift in the electric field density on the surface. This determines a change in the oscillation frequency of the electrons, generating different cross-sections for the optical properties, including absorption and scattering.

Recently, bimetallic NPs attracted large scientific attention, probably, more than pure metallic NPs [25,26,27,28]: in fact, bimetallic NPs are characterized by novel electronic, optical, catalytic or photocatalytic properties different from those of the monometallic counterparts. These properties arise from the combination of the properties corresponding to the two individual metals but also from a synergy between the two metals. The bimetallic NPs, typically, can present a random alloy structure or an alloy intermetallic compound structure or a Janus structure or a core–shell structure depending on the relative strengths of metal–metal bond, surface energies of the composing elements, relative atomic sizes, fabrication method [26]. In particular, core–shell bimetallic NPs (consisting of a shell made of one type of metal atoms surrounding a core made of other metal atoms) is particularly interesting since the resulting NPs properties can be controllably tuned simply by controlling the ratio of the core–shell sizes [25,26,27,28]. This last can be simply tuned, for example, during chemical-based synthetic approaches by controlling the process conditions. As a consequence, core–shell bimetallic NPs show technologically useful structure-dependent electronic, optical, catalytic, photocatalytic properties that are absent in the coincident monometallic nanoparticles. The optical properties of core–shell bimetallic NPs, in particular, are the subject of several investigations due to the occurrence of plasmonic properties and light-scattering properties, which are strongly determined by the interaction of the two constituting metals and by the size of the core and of the shell. In this sense, for example, the plasmonic absorption wavelength or the scattered light intensity can be controlled in a wide range by simply changing the nature of the metals or the size ratio between core and shell when fixed the constituting metals. Surface-enhanced Raman spectroscopy-based, photocatalytic and more other applications widely take advantage of this characteristic. Core–shell bimetallic NPs in which one of the metals is Au, Ag, or Cu are widely used in plasmonic-based (sensing and biosensing, energy conversion, biomedicine), catalytic-based, antimicrobial, electronic applications. Core–shell bimetallic NPs in which one of the metal is Pd or Pt is widely used in catalytic and electrocatalytic, hydrogen-storage, sensing applications. In this regard, several papers deal with the fabrication of AuAg and AgAu nanoparticles [29,30,31,32,33,34,35,36,37,38], CuAg and AgCu [38,39,40,41,42], AuPd and PdAu [43,44,45,46,47,48], AuPt and PtAu [49,50,51,52], PdPt and PtPd [53,54,55,56,57] core–shell NPs and with the study of their optical-based properties (and consequent potential applications) versus the core–shell size ratio. As an example, Cui et al. [57] investigated the absorption properties of bimetallic NPs formed by a Au core and a Pd or Pt shell by fixing the core size and changing the shell size. Considering, in particular, the Au-Pt core–shell NPs, with increasing Pt shell thickness, the Au plasmonic band (520 nm) decreases and disappears at the Pt shell thickness of 21 nm. Furthermore, a broad absorption band appears and red-shifts from about 550 to 650 nm when the thickness of the Pt shell increases from 21 to 40 nm. In addition, a broad absorption band in the UV region appears, which red-shifts by increasing shell thickness from 280 to 370 nm. This is a clear demonstration of the importance of investigating the impact of the core–shell sizes ratio on the optical properties of core–shell bimetallic NPs so as to set a general framework connecting these parameters for specific functional applications.

Recently, also, the computational approach to design specific geometries for light-scattering bimetallic core–shell nanoparticles got a renewed interest [27,58,59,60]. In fact, the calculations of the resulting optical properties of bimetallic core–shell nanoparticles are the starting point to identify promising candidate systems, with desired geometries, for cutting-edge technological applications as in photocatalysis [58], Raman spectroscopy, optical sensing, and photovoltaics [27]. The present work contributes to these ideas by analyzing the light-scattering properties of spherically bimetallic core–shell nanoparticles (which can be easily produced, for example, by chemical-based approaches) of selected metals with numerous potential technological applications.

On the basis of these considerations, this work reports, in particular, results concerning calculations, by using the Mie theory, of the angle-dependent light-scattering intensity (I(θ)) for some selected spherical core–shell bimetallic NPs, namely AuAg, CuAg, AuPd, AuPt, PdPt and their counterparts AgAu, AgCu, PdAu, PtAu, PtPd, due to their potential applications in several technological fields. Section 2 gives the basic theoretical concepts to treat the light-scattering phenomena from small homogeneous and layered spherical particles. Then, Section 3 reports the results for the calculations of the light-scattering intensity I(θ) and scattering efficiency Q_scatt_ for the spherical core–shell bimetallic NPs for a wide range of situations, i.e., by changing the core and shell sizes. As a consequence, for each combination of metals, the evolution of I(θ) and Q_scatt_ with the core and shell sizes are drawn, and comparisons between the various types of bimetallic core–shell NPs are also drawn at parity of core and shell sizes. For simplicity, the analysis is limited to spherical core–shell NPs so to use the Mie theory and perform exact analytical calculations. From the experimental point of view, non-spherical metal nanostructures (complex-morphology nanostructures) are also particularly interesting presenting plasmonic hot-spots in correspondence of apex-shaped geometries or double-bands plasmonic absorption as in nanorods [1,2,3,4,61]. In these systems, the interaction of electromagnetic radiation with metallic nanostructures is more complicated and can result in additional interesting effects towards technological applications. However, the results of the present work, even if simplified, can help in establishing the general effect of the core and shell sizes on the light-scattering properties of the core–shell bimetallic NPs essential to select preparation methods and conditions to prepare the NPs with desired structure appropriate to the application.

## 2. Basic Theory Concepts

A complete theory of the scattering and absorption of electromagnetic radiation by a homogeneous sphere was developed by Gustav Mie [18]. Mie’s approach relies on the expansion of the internal and scattered fields into a set of normal modes described by vector harmonics [62,63]. The quasi-static results valid for subwavelength spheres are then recovered by a power series expansion of the absorption and scattering coefficients and taking into account only the first term. Obviously, Mie’s theory is applicable only to spherical particles. Mie’s theory was also generalized to treat, analytically, the electromagnetic radiation scattering and absorption properties by multilayered spherical particles [62,63,64], which is the case to be applied regarding the interaction of spherical core–shell NPs with the radiation.

Shortly, following Small et al. [63], to characterize the scattering process of the electromagnetic radiation from a particle, the scattering cross-section σ is the main parameter. It is defined as the ratio between the total radiation scattered power to the radiation intensity, σ = W/I_0_ being I_0_ (energy/(area)(time)) the intensity of a plane electromagnetic wave impinging on the spherical particle and W (energy/time) the total (i.e., integrated over all directions) power of the wave scattered by the particle. Usually, what is experimentally measured is the scattering efficiency defined as Q_scatt_ = σ/πR^2^ being, simply, the scattering cross-section σ normalized to the geometrical section πR^2^ (area of a circle of radius R) of the spherical particle of radius R. Exploiting the definition of Q_scatt_, a dimensionless parameter, the electromagnetic radiation scattering properties of spherical particles with different sizes can be directly compared. Q_scatt_ can also exceed 1 for a particle since, in addition to scattering radiation incident on its geometrical cross-section, the particle also diffracts radiation at its edges so that it can behave as a larger particle than its geometrical cross-section. To calculate σ for a homogeneous spherical particle, Maxwell’s equations can be analytically solved considering a plane wave incident on the particle. Then, Q_scatt_ can be calculated. Similarly, Maxwell’s equations can be solved considering a layered spherical particle on which a plane electromagnetic wave is inciding: if the particle is formed by N layers (from 1, the core, to N, the outmost shell), with each layer having a radius R_i_ (R_1_ the radius of the core, R_N_ the thickness of the outmost shell) and refractive index n_i_ (n_1_ the refractive index of the core, n_N_ the refractive index of the outmost shell, n_b_ the refractive index of a matrix embedding the layered particle, n the refractive index of the medium that the wave is traveling), then the incident, scattered, and internal fields can be expanded as a superposition of vector spherical harmonics (thanks to the spherical symmetry). Calling $N_{\mathrm{mp}}\left(k_{0}\mathrm{nr},\theta ,\phi \right)$ the Hankel function describing the waves radiating outwards from the origin of the reference system (where the particle is located) and $N_{\mathrm{mp}}^{*}\left(k_{0}\mathrm{nr},\theta ,\phi \right)$ the Hankel function describing waves converging inward toward the origin (being k_0_ = 2π/c (c the light velocity in vacuum), * a superscript indicating the complex conjugation, r, θ, and Φ the spherical coordinates) then, the plane wave field incident on the particle and the field scattered by the particle can be expressed, respectively, as [63]:

(1) $$E_{\mathrm{inc}}=\frac{1}{2}\overset{2}{\underset{p=1}{\sum }}\overset{\infty }{\underset{m=1}{\sum }}q_{\mathrm{mp}}\left[N_{\mathrm{mp}}\left(k_{0}n_{b}r,\theta ,\phi \right)+N_{\mathrm{mp}}^{*}\left(k_{0}n_{b}r,\theta ,\phi \right)\right]$$

(2) $$E_{\mathrm{scatt}}=\overset{2}{\underset{p=1}{\sum }}\overset{\infty }{\underset{m=1}{\sum }}q_{\mathrm{mp}}a_{\mathrm{mp}}N_{\mathrm{mp}}\left(k_{0}n_{b}r,\theta ,\phi \right)$$

where q_mp_ = −[i^n + p^ (2n + 1)/n(n + 1)], the a_mp_ is proportional to a scattering amplitude, p indicates the polarization of the wave (p = 1 for transverse magnetic waves, p = 2 for transverse electric waves), m is the order of the Hankel function of the first kind describing the radial dependence of the vector spherical harmonics. The scattering cross-section σ can be calculated by determining the coefficients {a_mp_}. Therefore, the problem is shifted in the determination of these coefficients. To this end, the field in the jth layer of the particle can be expressed as [63]:

(3) $$E_{\mathrm{int}}^{j}=\sum_{p=1}^{2}\sum_{m=1}^{\infty }q_{\mathrm{mp}}\left[u_{\mathrm{mp}}^{j}N_{\mathrm{mp}}\left(k_{0}n_{j}r,\theta ,\phi \right)+v_{\mathrm{mp}}^{j}N_{\mathrm{mp}}^{*}\left(k_{0}n_{j}r,\theta ,\phi \right)\right],\;2\;\leq \;j\;\leq \;N$$

(4) $$E_{\mathrm{int}}^{1}=\overset{2}{\underset{p=1}{\sum }}\overset{\infty }{\underset{m=1}{\sum }}q_{\mathrm{mp}}\left[u_{\mathrm{mp}}^{1}N_{\mathrm{mp}}\left(k_{0}n_{1}r,\theta ,\phi \right)+N_{\mathrm{mp}}^{*}\left(k_{0}n_{1}r,\theta ,\phi \right)\right]$$

being $u_{\mathrm{mp}}^{j}$ and $v_{\mathrm{mp}}^{j}$ coefficients characterizing the outgoing and incoming fields. Equations (3) and (4) requires a number of boundary conditions, from which the determination of the coefficients {a_mp_} arise: first of all, at the core of the particle, the condition $u_{\mathrm{mp}}^{1}=v_{\mathrm{mp}}^{j}$ holds (this means that the amplitudes of the incoming and outgoing fields must be equal at the center of the particle); the additional boundary conditions concern the fact that the transverse components of the electric and magnetic fields are continuous across the boundary between layers j and j + 1 (1 < j < N−1) and between layer N and the surrounding matrix. As a consequence of these conditions, a set of 2 N equations arises, which can be solved to determine the set of coefficients {a_mp_} and, then, the scattering cross-section σ and, then, the scattering efficiency Q_scatt_.

This approach is exploited by algorithms used in various software to carry out the calculations [65], for example, pySCATMECH, LORENTZ-MIE SCATTERING, PYSHS, STRATIFY, MIEPYTHON, PYMIESCATT, MENP, SCATLAB, etc. In particular, in the present work, the ScatLab 1.2.111 software [66] is used to calculate, in particular, the angle-dependent intensities (I(θ)) and the scattering efficiency (Q_scatt_) for the bimetallic spherical core–shell particles (AuAg, CuAg, AuPd, AuPt, PdPt and their counterparts AgAu, AgCu, PdAu, PtAu, PtPd) by changing the size of the core and of the shell and by fixing the wavelength of the incident electromagnetic wave to λ = 550 nm (the center of the visible spectrum, in view of visible-light-induced phenomena and visible light-based applications). ScatLab is software developed to perform electromagnetic scattering simulations mainly based on classical Mie theory solution. It is designed to meet windows type guidelines. The computation capabilities of ScatLab rely on the possibility to calculate, as main examples, scattered intensity polar diagrams for coated and uncoated spherical particles, scattered intensity versus radius graphs for homogeneous spherical particles, polarization rate versus radius graph for homogeneous spherical particles, extinction, scattering and backscattering cross-section graphs, polarization rate versus damping rate graph, angle depolarization graphs, near field imaging for homogeneous spherical particles, near field average scattered intensity versus radius graphs for homogeneous spherical particles, Lorentz and Drude dielectric function implementation for refractive index calculation, and more other. As generally described above, the ScatLab software is one type of calculator (based on the Mie theory), which considers an incident plane wave as represented by an infinite combination of spherical harmonics. Their amplitudes depend on the polarization and the direction of the incident wave and are given in general cases by analytical formulae. The advantage of such representation is in that each such harmonics is scattered as a single spherical outgoing harmonics, which amplitude depends on the particle radius and refractive indices and is prescribed by coefficient given by an analytical expression. Since each scattered harmonics propagates independently, the total scattered power is found as the sum of particular powers in all scattered harmonics. The main limits of the ScatLab calculator arise from the basic approximations for which the Mie theory holds, mainly, Mie theory gives appreciable results for quite larger particles (having size parameter comparable to the wavelength of incident light). This is because the theory cannot accommodate well for very small particles, especially particles approximately 1/10th of λ. So, for particles below the size of nearly 50 nm, Mie’s theory will not give good results when considering impinging visible light. In addition, the solutions of the calculations are dependent on the specific boundary conditions under which the program operates. Generally, the following conditions are imposed: (a) interface conditions on the boundary between the spherical particle and the environment (which allow us to relate the expansion coefficients of the incident, internal, and scattered fields); (b) the condition that the solution is bounded at the origin; (c) for a scattered field, the asymptotes at infinity corresponds to a diverging spherical wave. Values commonly calculated by software using Mie theory, as ScatLab, include efficiency coefficients for extinction, scattering, and absorption. The solutions solve for an infinite harmonic series and provide as output the calculation of the scattering phase function, extinction, scattering, and absorption efficiencies. These efficiency coefficients are ratios of the cross-section of the respective process to the particle area. The dependence of the scattering cross-section on the wavelength and the contribution of specific resonances strongly depends on the particle material. For example, for a Au particle with a radius of 100 nm, the contribution of the electric dipole to scattering predominates in the optical range, while for a Si particle, there are pronounced magnetic dipole and quadrupole resonances. For metal particles, the peak visible in the scattering cross-section is the localized plasmon resonance. In the limit of small particles or long wavelengths, the electric dipole contribution dominates in the scattering cross-section. Hence, it is of paramount importance the selection of the values for the real part and imaginary part of the refractive index of the material composing the particle for each analyzed wavelength to obtain reliable results. Overall, however, the ScatLab software was widely used the calculate the optical properties of several typologies of spherical single-component or multilayered particles with excellent results since in agreement with the experimental results within the range for which the Mie theory holds and for which the experimental conditions adheres to the validity hypothesis for the theory [63,67,68,69,70].

In particular, we exploit ScatLab’s capabilities to calculate the light-scattering properties of the spherical bimetallic core–shell NPs made by AuAg, AgAu, CuAg, AgCu, AuPd, PdAu, AuPt, PtAu, PdPt, PtPd for various combinations of the core radius and shell width: hence, within the capabilities and limits of the ScatLab software, the additional scientific inside of the present work relies on in the application of freely available software to functional nanomaterials with potentially interesting applications and in the setting of a general framework connecting the NPs geometry to their light-scattering characteristics. Therefore, our work is in line with the computational design of the best geometries for the core–shell bimetallic nanoparticles for desired optical properties for specific applications.

In order to perform the calculations, the ScatLab software requires, as input parameters, values for the real part, n, and imaginary part, k, of the refractive index of the materials composing the particle and of the matrix where the particle is embedded (and corresponding to the chosen wavelength of the incident electromagnetic radiation), and values for the particle core radius R and particle shell width d. Regarding the metals here investigated, the values for n and k used for the calculations are reported in Table 1 (for λ = 550 nm) as extracted by ref [71]. The particles are supposed to be placed in the air so that n = 1 and k = 0 for the matrix embedding the particles.

## 3. Calculations and Discussion

**Figure 1:**
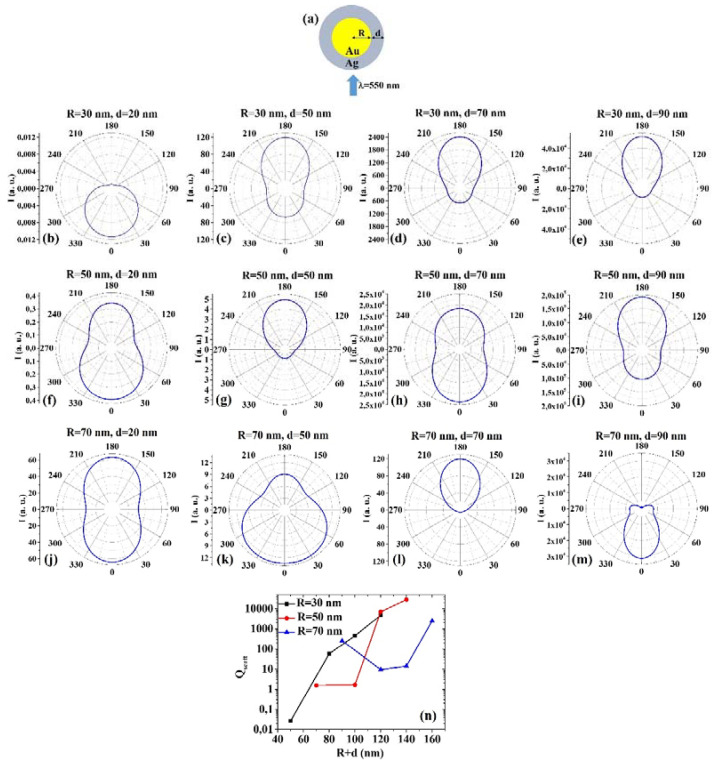
(**a**) Scheme of the structure of the simulated Au/Ag core/shell spherical particle with core radius R and shell width d and electromagnetic radiation of wavelength λ = 550 nm impinging on the particle from 0°. (**b**–**m**) Calculated polar diagrams for the intensity of the scattered light from the Au/Ag core/shell spherical particle changing the Au core radius R and the Ag shell width: (**b**–**e**) R = 30 nm and d = 20 nm (**b**), d = 50 nm (**c**), d = 70 nm (**d**), d = 90 nm (**e**); (**f**–**i**) R = 50 nm and d = 20 nm (**f**), d = 50 nm (**g**), d = 70 nm (**h**), d = 90 nm (**i**); (**j**–**m**) R = 70 nm and d = 20 nm (**j**), d = 50 nm (**k**), d = 70 nm (**l**), d = 90 nm (**m**). (**n**) Calculated scattering efficiency for the light (wavelength λ = 550 nm) scattering process of the Au/Ag spherical particle fixing the Au core diameter (R = 30 nm black dots, R = 50 nm red dots, R = 70 nm blue dots) and increasing the Ag shell width d from 20 nm to 90 nm.

The ScatLab software is now used to perform electromagnetic scattering simulations for the spherical bimetallic core–shell NPs: in particular, an electromagnetic plane wave of wavelength λ = 550 nm is supposed to impinge from 0° on the single NP, which is located in the origin of a reference system. Then, the ScatLab software is used to calculate the angular-dependent intensity I(θ) of the scattered electromagnetic wave and the scattering efficiency Q_scatt_. This is done for the spherical bimetallic core–shell NPs made by AuAg, AgAu, CuAg, AgCu, AuPd, PdAu, AuPt, PtAu, PdPt, PtPd for various combinations of the core radius R (30, 50, 70 nm) and shell width d (20, 50, 70, 90 nm). The other input parameters are the values of n and k, as reported in Table 1. In each case, the results for the calculations of I(θ) are reported in polar diagrams, and the results for the calculations of Q_scatt_ are reported in plots expressing the evolution of Q_scatt_ for each couple of metals when fixed R and increasing d. The results are reported from Figure 1 to Figure 10. In particular, (1) AuAg: Figure 1a shows the picture of the structure of the Au/Ag core/shell spherical particle for which the simulations are performed with R the core radius and d shell width. The electromagnetic radiation of wavelength λ = 550 nm impinges on the particle from 0°. In addition, Figure 1b–m presents the calculated polar diagrams for the intensity of the scattered light from the Au/Ag core/shell spherical particle changing the Au Tablecore radius R and the Ag shell width (i.e., fixing R to 30 nm or 50 nm or 70 nm and increasing d from 20 nm to 90 nm). Finally, Figure 1n presents the calculated scattering efficiency values for the light (wavelength λ = 550 nm) scattering process of the Au/Ag spherical particle fixing the Au core diameter (R = 30 nm black dots, R = 50 nm red dots, R = 70 nm blue dots) and increasing the Ag shell width d from 20 nm to 90 nm.

In this case, we observe, in particular, that fixing R = 30 nm, the intensity I of the scattered radiation is maximum for θ = 0° when d =2 0 nm, while I is maximum for θ = 180° when d = 50 nm, d = 70 nm, d = 90 nm. Fixing R = 50 nm, I is maximum for θ = 0° when d = 20 nm and d = 70 nm, maximum for θ = 180° when d = 50 nm and 90 nm. Fixing R = 70 nm, I is maximum for θ = 0° when d = 20 nm even if for θ = 180° the intensity value is very similar (a little bit lower). For R = 70 nm and d = 50 nm and d = 90 nm, I is maximum when θ = 0°, while for R = 70 nm and d = 70 nm I is maximum when θ = 180°. The scattering efficiency, Q_scatt_, when fixed R = 30 nm, increases monotonically by increasing d from 20 nm to 90 nm; when fixed R = 50 nm, Q_scatt_ is about constant for d increasing from 20 nm to 50 nm and, then, monotonically increases by increasing d from 50 nm to 90 nm; when fixed R = 70 nm, Q_scatt_ decreases by increasing d from 20 to 50 nm and, then, monotonically increases by increasing d from 50 nm to 90 nm. The lowest scattering efficiency is obtained for R = 30 nm and d = 20 nm; the highest scattering efficiency is obtained for R = 50 nm and d = 90 nm. Hence, in this case, the best geometry to achieve the highest light-scattering efficiency for applications taking advantage of these properties is that for which R = 50 nm and d = 90 nm.

**Figure 2:**
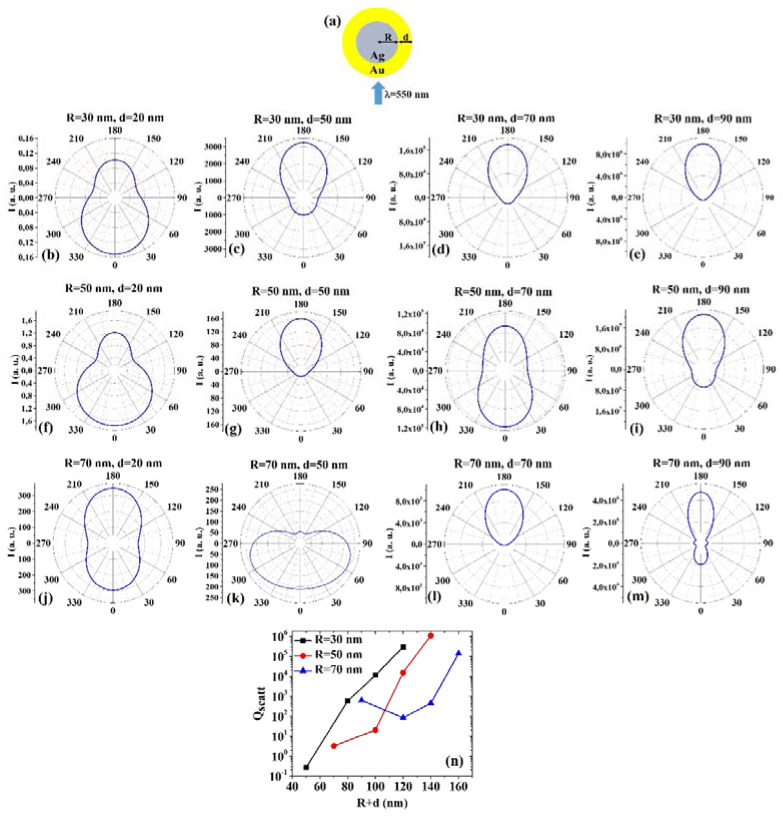
(**a**) Scheme of the structure of the simulated Ag/Au core/shell spherical particle with core radius R and shell width d and electromagnetic radiation of wavelength λ = 550 nm impinging on the particle from 0°. (**b**–**m**) Calculated polar diagrams for the intensity of the scattered light from the Ag/Au core/shell spherical particle changing the Ag core radius R and the Au shell width: (**b**–**e**) R = 30 nm and d = 20 nm (**b**), d = 50 nm (**c**), d = 70 nm (**d**), d = 90 nm (**e**); (**f**–**i**) R = 50 nm and d = 20 nm (**f**), d = 50 nm (**g**), d = 70 nm (**h**), d = 90 nm (**i**); (**j**–**m**) R = 70 nm and d = 20 nm (**j**), d = 50 nm (**k**), d = 70 nm (**l**), d = 90 nm (**m**). (**n**) Calculated scattering efficiency for the light (wavelength λ = 550 nm) scattering process of the Ag/Au spherical particle fixing the Ag core diameter (R = 30 nm black dots, R = 50 nm red dots, R = 70 nm blue dots) and increasing the Au shell width d from 20 nm to 90 nm.

(2) AgAu: Figure 2a shows the picture of the structure of the Ag/Au core/shell spherical particle for which the simulations are performed with R the core radius and d shell width. The electromagnetic radiation of wavelength λ = 550 nm impinges on the particle from 0°. In addition, Figure 2b–m presents the calculated polar diagrams for the intensity of the scattered light from the Ag/Au core/shell spherical particle changing the Ag core radius R and the Au shell width (i.e., fixing R to 30 nm or 50 nm or 70 nm and increasing d from 20 nm to 90 nm). Finally, Figure 2n presents the calculated scattering efficiency values for the light (wavelength λ = 550 nm) scattering process of the Au/Ag spherical particle fixing the Au core diameter (R = 30 nm black dots, R = 50 nm red dots, R = 70 nm blue dots) and increasing the Ag shell width d from 20 nm to 90 nm.

In this case, we observe, in particular, that fixing R = 30 nm, the intensity I of the scattered radiation is maximum for θ = 0° when d = 20 nm, while I is maximum for θ = 180° when d = 50 nm, d = 70 nm, d = 90 nm. Fixing R = 50 nm, I is maximum for θ = 0° when d = 20 nm and d = 70 nm, maximum for θ = 180° when d = 50 nm and 90 nm. Fixing R = 70 nm, I is maximum for θ = 180° when d = 20 nm, d = 70 nm and d = 90 nm, while, interestingly, I is maximum for 60° < θ < 75° and 285° < θ < 300°. The scattering efficiency, Q_scatt_, when fixed R = 30 nm, increases monotonically by increasing d from 20 nm to 90 nm; when fixed R = 50 nm, Q_scatt_ is about constant by increasing d from 20 nm to 50 nm and, then, monotonically increases by increasing d from 50 nm to 90 nm; when fixed R = 70 nm, Q_scatt_ decreases by increasing d from 20 nm to 50 nm and, then, monotonically increases by increasing d from 50 nm to 90 nm. The lowest scattering efficiency is obtained for R = 30 nm and d = 20 nm; the highest scattering efficiency is obtained for R = 50 nm and d = 90 nm. Hence, in this case, the best geometry to achieve the highest light-scattering efficiency for applications taking advantage of these properties is that for which R = 50 nm and d = 90 nm.

Comparing, for example, Figure 1b and Figure 2b, we can observe that the Ag/Au particle is more effective in scattering the radiation at 0° than the Au/Ag particle; comparing Figure 1k and Figure 2k, we can observe that the Au/Ag particle is more effective in scattering the radiation at 0° than the Ag/Au particle; comparing Figure 1m and Figure 2m, we can observe that the Au/Ag particle is more effective in scattering the radiation at 0° than the Ag/Au particle and that the Ag/Au particle is more effective in scattering the radiation at 180° than the Au/Ag particle. Hence, generally, the Ag/Au geometry has the highest scattering efficiency and, so this is the favorite geometry in applications taking advantage of this property.

**Figure 3:**
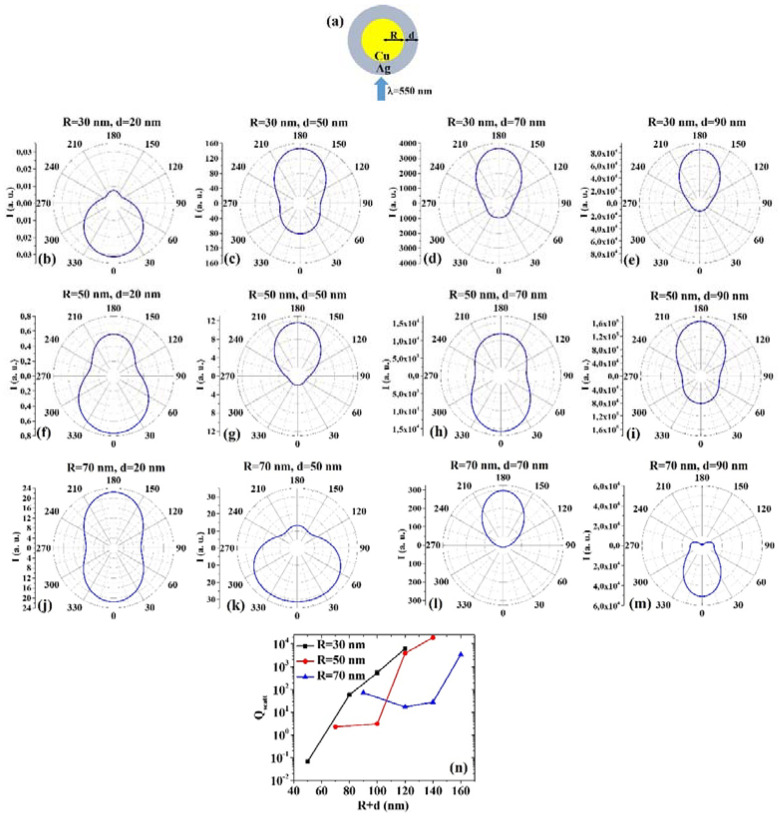
(**a**) Scheme of the structure of the simulated Cu/Ag core/shell spherical particle with core radius R and shell width d and electromagnetic radiation of wavelength λ = 550 nm impinging on the particle from 0°. (**b**–**m**) Calculated polar diagrams for the intensity of the scattered light from the Cu/Ag core/shell spherical particle changing the Cu core radius R and the Ag shell width: (**b**–**e**) R = 30 nm and d = 20 nm (**b**), d = 50 nm (**c**), d = 70 nm (**d**), d = 90 nm (**e**); (**f**–**i**) R = 50 nm and d = 20 nm (**f**), d = 50 nm (**g**), d = 70 nm (**h**), d = 90 nm (**i**); (**j**–**m**) R = 70 nm and d = 20 nm (**j**), d = 50 nm (**k**), d = 70 nm (**l**), d = 90 nm (**m**). (**n**) Calculated scattering efficiency for the light (wavelength λ = 550 nm) scattering process of the Cu/Ag spherical particle fixing the Cu core diameter (R = 30 nm black dots, R = 50 nm red dots, R = 70 nm blue dots) and increasing the Ag shell width d from 20 nm to 90 nm.

(3) CuAg: Figure 3a shows the picture of the structure of the Cu/Ag core/shell spherical particle for which the simulations are performed with R the core radius and d shell width. The electromagnetic radiation of wavelength λ = 550 nm impinges on the particle from 0°. In addition, Figure 3b–m presents the calculated polar diagrams for the intensity of the scattered light from the Cu/Ag core/shell spherical particle changing the Cu core radius R and the Ag shell width (i.e., fixing R to 30 nm or 50 nm or 70 nm and increasing d from 20 nm to 90 nm). Finally, Figure 3n presents the calculated scattering efficiency values for the light (wavelength λ = 550 nm) scattering process of the Cu/Ag spherical particle fixing the Au core diameter (R = 30 nm black dots, R = 50 nm red dots, R = 70 nm blue dots) and increasing the Ag shell width d from 20 nm to 90 nm.

In this case, we observe, in particular, that fixing R = 30 nm, the intensity I of the scattered radiation is maximum for θ = 0° when d = 20 nm and I is maximum for θ = 180° when d = 50 nm, d = 70 nm, d =9 0 nm. Setting R=50 nm, I is maximum for θ = 0° when d = 20 nm and d = 70 nm, maximum for θ = 180° when d = 50 nm and 90 nm. Fixing R = 70 nm, I is maximum for θ = 180° when d = 20 nm even if for θ = 0° the intensity values are very similar (a little bit lower). For R = 70 nm and d = 50 nm and d = 90 nm, I is maximum when θ = 0°, while for R = 70 nm and d = 70 nm I is maximum when θ = 180°. The scattering efficiency, Q_scatt_, increases monotonically by increasing d from 20 nm to 90 nm for R = 30 nm; when fixed R = 50 nm, Q_scatt_ is about constant by increasing d from 20 nm to 50 nm and, then, monotonically increases by increasing d from 50 nm to 90 nm; setting R = 70 nm, Q_scatt_ decreases by increasing d from 20 to 50 nm and, then, monotonically increases by increasing d from 50 nm to 90 nm. The lowest scattering efficiency is obtained for R = 30 nm and d = 20 nm; the highest scattering efficiency is obtained for R = 50 nm and d = 90 nm. Hence, the condition R = 50 nm and d = 90 nm establishes the geometry with the highest scattering efficiency for selected applications.

**Figure 4:**
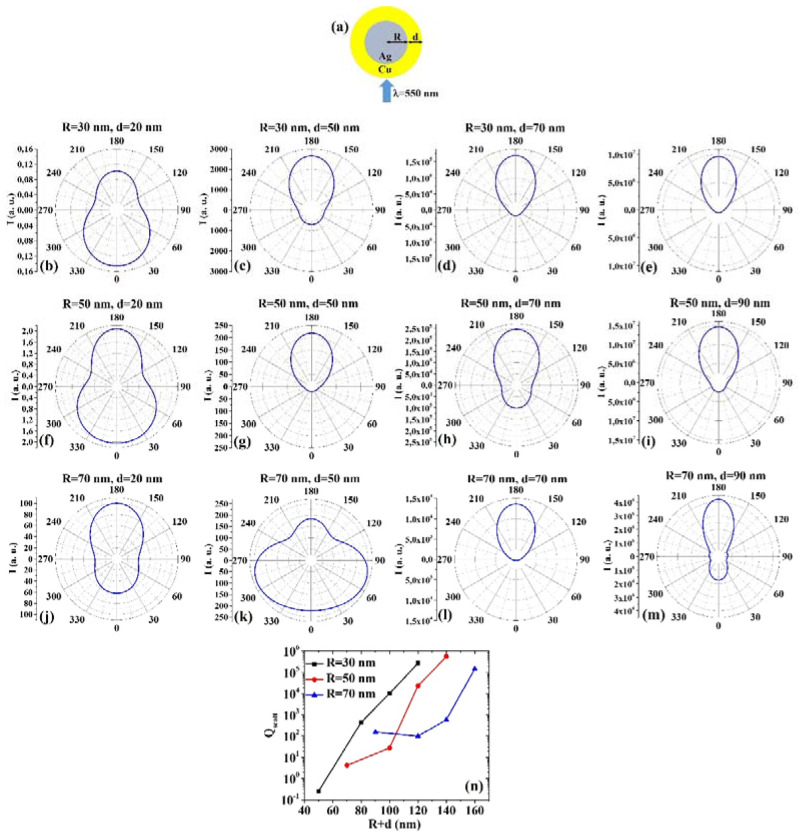
(**a**) Scheme of the structure of the simulated Ag/Cu core/shell spherical particle with core radius R and shell width d and electromagnetic radiation of wavelength λ = 550 nm impinging on the particle from 0°. (**b**–**m**) Calculated polar diagrams for the intensity of the scattered light from the Ag/Cu core/shell spherical particle changing the Ag core radius R and the Cu shell width: (**b**–**e**) R = 30 nm and d = 20 nm (**b**), d = 50 nm (**c**), d = 70 nm (**d**), d = 90 nm (**e**); (**f**–**i**) R = 50 nm and d = 20 nm (**f**), d = 50 nm (**g**), d = 70 nm (**h**), d = 90 nm (**i**); (**j**–**m**) R = 70 nm and d = 20 nm (**j**), d = 50 nm (**k**), d = 70 nm (**l**), d = 90 nm (**m**). (**n**) Calculated scattering efficiency for the light (wavelength λ = 550 nm) scattering process of the Ag/Cu spherical particle fixing the Ag core diameter (R = 30 nm black dots, R = 50 nm red dots, R = 70 nm blue dots) and increasing the Cu shell width d from 20 nm to 90 nm.

(4) AgCu: Figure 4a reports the picture of the structure of the Ag/Cu core/shell spherical particle for which the simulations are performed with R the core radius and d shell width. The electromagnetic radiation of wavelength λ = 550 nm impinges on the particle from 0°. In addition, Figure 4b–m presents the calculated polar diagrams for the intensity of the scattered light from the Ag/Cu core/shell spherical particle changing the Ag core radius R and the Cu shell width (i.e., fixing R to 30 nm or 50 nm or 70 nm and increasing d from 20 nm to 90 nm). Finally, Figure 4n presents the calculated scattering efficiency values for the light (wavelength λ = 550 nm) scattering process of the Ag/Cu spherical particle fixing the Au core diameter (R = 30 nm black dots, R = 50 nm red dots, R = 70 nm blue dots) and increasing the Ag shell width d from 20 nm to 90 nm.

In this case, we observe, in particular, that fixing R = 30 nm, the intensity I of the scattered radiation is maximum for θ = 0° when d = 20 nm, while I is maximum for θ = 180° when d = 50 nm, d = 70 nm, d = 90 nm. Setting R = 50 nm, I is maximum for θ = 180° when d = 20 nm even if for θ = 0° the intensity value is very similar (a little bit lower), and maximum for θ = 180° when d = 50 nm, d = 70 nm and d = 90 nm. Fixing R = 70 nm, I is maximum for θ=180° when d=20 nm, d=70 nm and d=90 nm. However, in this case, for R = 70 nm and d = 50 nm, I is maximum for 60° < θ < 75° and 285° < θ < 300°. Setting R = 30 nm or R = 50 nm, the scattering efficiency, Q_scatt_, increases monotonically by increasing d from 20 nm to 90 nm; when fixed R = 70 nm, Q_scatt_ decreases by increasing d from 20 to 50 nm and, then, monotonically increases by increasing d from 50 nm to 90 nm. The lowest scattering efficiency is obtained for R = 30 nm and d = 20 nm, and the highest scattering efficiency is obtained for R = 50 nm and d = 90 nm. Hence, the condition R = 50 nm and d = 90 nm establishes the geometry with the highest scattering efficiency in vie of application exploiting this property.

Comparing, for example, Figure 3b and Figure 4b, we can observe that the Ag/Cu particle is more effective in scattering the radiation at 180° than the Cu/Ag particle; comparing Figure 3h and Figure 4h, we can observe that the Cu/Ag particle is more effective in scattering the radiation at 0° than the Ag/Cu particle; comparing Figure 3m and Figure 4m, we can observe that the Cu/Ag particle is more effective in scattering the radiation at 0° than the Ag/Cu particle and that the Ag/Cu particle is more effective in scattering the radiation at 180° than the Cu/Ag particle. Comparing the plots of the scattering efficiency, however, we can conclude, generally, that the Ag/Cu geometry has the highest efficiency, so that this would be the favored geometry in applications involving this process.

**Figure 5:**
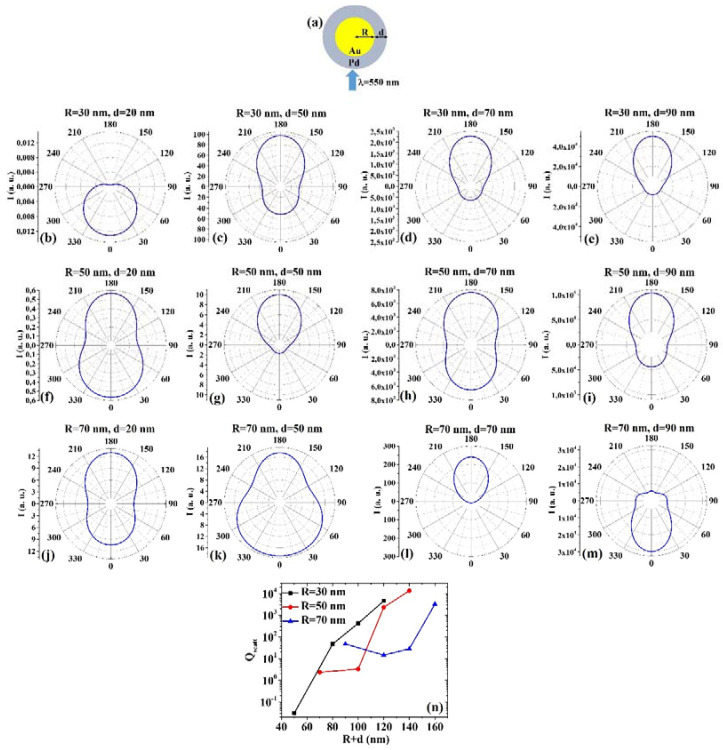
(**a**) Scheme of the structure of the simulated Au/Pd core/shell spherical particle with core radius R and shell width d and electromagnetic radiation of wavelength λ = 550 nm impinging on the particle from 0°. (**b**–**m**) Calculated polar diagrams for the intensity of the scattered light from the Au/Pd core/shell spherical particle changing the Au core radius R and the Pd shell width: (**b**–**e**) R = 30 nm and d = 20 nm (**b**), d = 50 nm (**c**), d = 70 nm (**d**), d = 90 nm (**e**); (**f**–**i**) R = 50 nm and d = 20 nm (**f**), d = 50 nm (**g**), d = 70 nm (**h**), d = 90 nm (**i**); (**j**–**m**) R = 70 nm and d = 20 nm (**j**), d = 50 nm (k), d = 70 nm (**l**), d = 90 nm (**m**). (**n**) Calculated scattering efficiency for the light (wavelength λ = 550 nm) scattering process of the Au/Pd spherical particle fixing the Au core diameter (R = 30 nm black dots, R = 50 nm red dots, R = 70 nm blue dots) and increasing the Pd shell width d from 20 nm to 90 nm.

(5) AuPd: Figure 5a presents the picture of the structure of the Au/Pd core/shell spherical particle for which the simulations are performed with R the core radius and d shell width. The electromagnetic radiation of wavelength λ = 550 nm impinges on the particle from 0°. In addition, Figure 5b–m shows the calculated polar diagrams for the intensity of the scattered light from the Au/Pd core/shell spherical particle changing the Au core radius R and the Pd shell width (i.e., fixing R to 30 nm or 50 nm or 70 nm and increasing d from 20 nm to 90 nm). Finally, Figure 5n presents the calculated scattering efficiency values for the light (wavelength λ = 550 nm) scattering process of the Au/Pd spherical particle fixing the Au core diameter (R = 30 nm black dots, R = 50 nm red dots, R = 70 nm blue dots) and increasing the Pd shell width d from 20 nm to 90 nm.

In this case, we observe, in particular, that fixing R = 30 nm, the intensity I of the scattered radiation is maximum for θ = 0° when d = 20 nm, while I is maximum for θ = 180° when d = 50 nm, d = 70 nm, d = 90 nm. Fixing R = 50 nm, I is maximum for θ = 0° when d = 20 nm even if for θ = 180° the intensity value is very similar (a little bit lower), and maximum for θ = 180° when d = 50 nm, d = 70 nm and d = 90 nm. Fixing R = 70 nm, I is maximum for θ = 180° when d = 20 nm and d = 70 nm, while it is maximum for θ = 0° when d = 50 nm and d = 90 nm. In particular, I only slightly decreases from the maximum value for θ increasing from 0° to 60° and from 300° to 360° when R = 70 nm and d = 50 nm. The scattering efficiency, Q_scatt_, when fixed R = 30 nm, increases monotonically by increasing d from 20 nm to 90 nm; when fixed R = 50 nm, Q_scatt_ is about constant by increasing d from 20 nm to 50 nm and, then, monotonically increases by increasing d from 50 nm to 90 nm; when fixed R = 70 nm, Q_scatt_ decreases by increasing d from 20 to 50 nm and, then, monotonically increases by increasing d from 50 nm to 90 nm. The lowest scattering efficiency is obtained for R = 30 nm and d = 20 nm; the highest scattering efficiency is obtained for R = 50 nm and d = 90 nm so that this geometry is the best one in application exploiting light-scattering.

**Figure 6:**
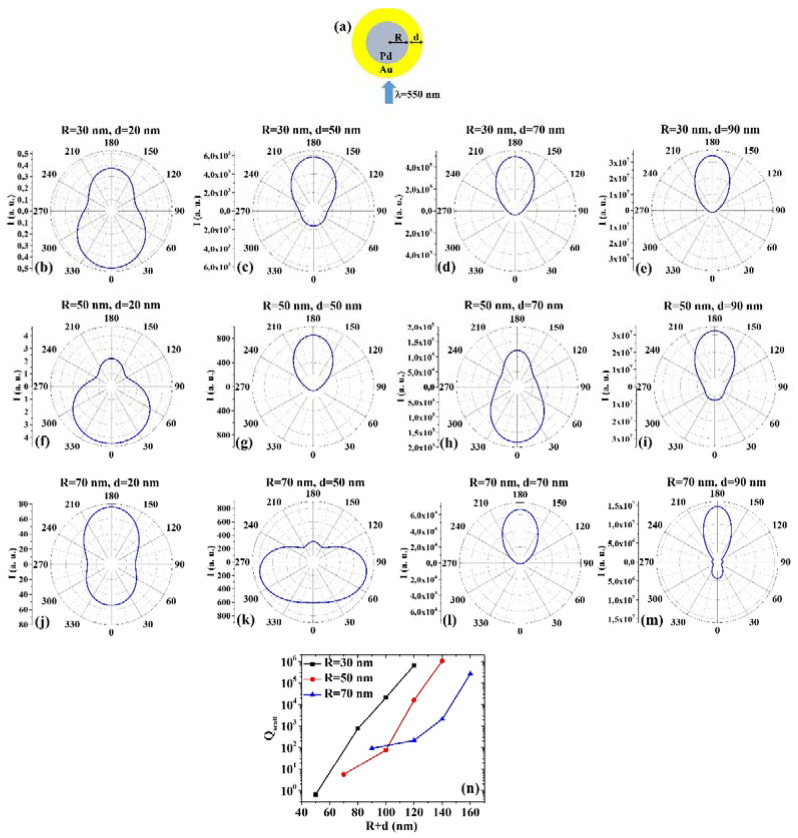
(**a**) Scheme of the structure of the simulated Pd/Au core/shell spherical particle with core radius R and shell width d and electromagnetic radiation of wavelength λ = 550 nm impinging on the particle from 0°. (**b**–**m**) Calculated polar diagrams for the intensity of the scattered light from the Pd/Au core/shell spherical particle changing the Pd core radius R and the Au shell width: (**b**–**e**) R = 30 nm and d = 20 nm (**b**), d = 50 nm (**c**), d = 70 nm (**d**), d = 90 nm (**e**); (**f**–**i**) R = 50 nm and d = 20 nm (**f**), d = 50 nm (**g**), d = 70 nm (**h**), d = 90 nm (**i**); (**j**–**m**) R = 70 nm and d = 20 nm (**j**), d = 50 nm (**k**), d = 70 nm (**l**), d = 90 nm (**m**). (**n**) Calculated scattering efficiency for the light (wavelength λ = 550 nm) scattering process of the Pd/Au spherical particle fixing the Pd core diameter (R = 30 nm black dots, R = 50 nm red dots, R = 70 nm blue dots) and increasing the Au shell width d from 20 nm to 90 nm.

(6) PdAu: Figure 6a presents the picture of the structure of the Pd/Au core/shell spherical particle for which the simulations are performed with R the core radius and d shell width. The electromagnetic radiation of wavelength λ = 550 nm impinges on the particle from 0°. In addition, Figure 6b–m shows the calculated polar diagrams for the intensity of the scattered light from the Pd/Au core/shell spherical particle changing the Pd core radius R and the Au shell width (i.e., fixing R to 30 nm or 50 nm or 70 nm and increasing d from 20 nm to 90 nm). Finally, Figure 6n presents the calculated scattering efficiency values for the light (wavelength λ = 550 nm) scattering process of the Pd/Au spherical particle fixing the Pd core diameter (R = 30 nm black dots, R = 50 nm red dots, R = 70 nm blue dots) and increasing the Au shell width d from 20 nm to 90 nm.

In this case, we observe, in particular, that fixing R = 30 nm, the intensity I of the scattered radiation is maximum for θ = 0° when d = 20 nm, while I is maximum for θ = 180° when d = 50 nm, d = 70 nm, d = 90 nm. Fixing R = 50 nm, I is maximum for θ = 0° when d = 20 nm and d = 70 nm, while I is maximum for θ = 180° when d = 50 nm and d = 90 nm. Fixing R = 70 nm, I is maximum for θ = 180° when d = 20 nm, d = 70 nm, and d = 90 nm. Interestingly, when R = 70 nm and d = 50 nm, I is maximum around 75° and around 285°. The scattering efficiency, Q_scatt_, monotonically increases monotonically for each fixed R (30, 50, 70 nm) by increasing d from 20 nm to 90 nm. The lowest scattering efficiency is obtained for R = 30 nm and d = 20 nm; the highest scattering efficiency is obtained for R = 50 nm and d = 90 nm, so that this is the best geometry for light-scattering-based applications.

Comparing, for example, Figure 5b and Figure 6b, we can observe that the Pd/Au particle is more effective in scattering the radiation at 180° than the Au/Pd particle; comparing Figure 5f and Figure 6f, we can observe that the Au/Pd particle is more effective in scattering the radiation at 180° than the Pd/Au particle; comparing Figure 5h and Figure 6h, we can observe that the Au/Pd particle is more effective in scattering the radiation at 180° than the Pd/Au particle; comparing Figure 5k and Figure 6k, we can observe that the Au/Pd particle is more effective in scattering the radiation both at 0° and 180° than the Pd/Au particle; comparing Figure 5m and Figure 6m, we can observe that the Au/Pd particle is more effective in scattering the radiation at 0° than the Pd/Au particle and the Pd/Au particle is more effective in scattering the radiation at 180° than the Au/Pd particle. However, comparing the plots of the scattering efficiency, it appears that, generally, the Pd/Au geometry is more efficient in scattering light than the Au/Pd one.

**Figure 7:**
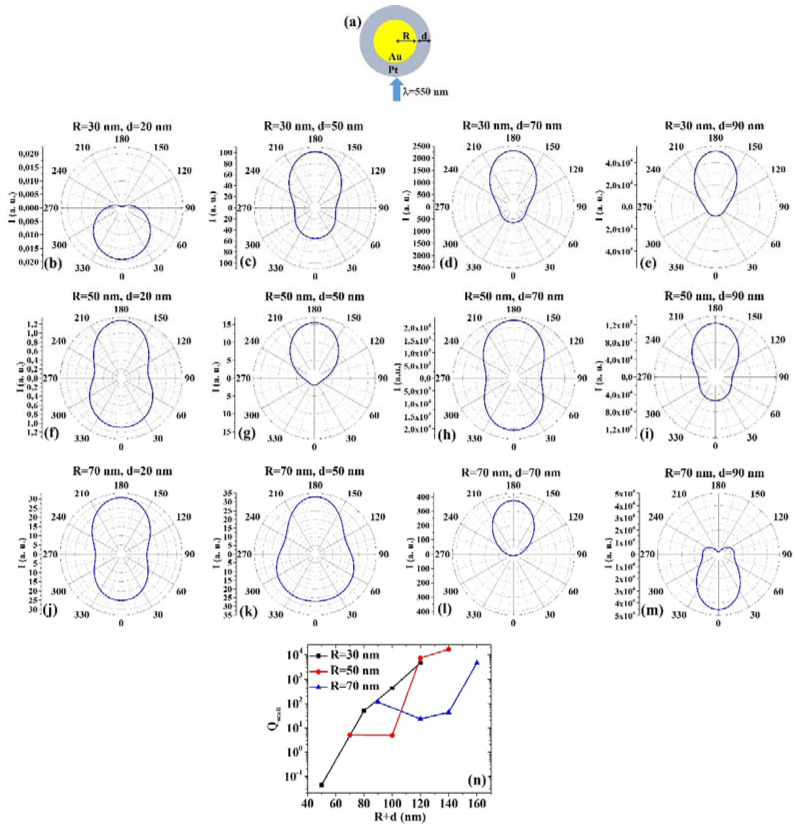
(**a**) Scheme of the structure of the simulated Au/Pt core/shell spherical particle with core radius R and shell width d and electromagnetic radiation of wavelength λ = 550 nm impinging on the particle from 0°. (**b**–**m**) Calculated polar diagrams for the intensity of the scattered light from the Au/Pt core/shell spherical particle changing the Au core radius R and the Pt shell width: (b–e) R = 30 nm and d = 20 nm (**b**), d = 50 nm (**c**), d = 70 nm (**d**), d = 90 nm (**e**); (**f**–**i**) R = 50 nm and d = 20 nm (**f**), d = 50 nm (**g**), d = 70 nm (**h**), d = 90 nm (**i**); (**j**–**m**) R = 70 nm and d = 20 nm (**j**), d = 50 nm (**k**), d = 70 nm (**l**), d = 90 nm (**m**). (**n**) Calculated scattering efficiency for the light (wavelength λ = 550 nm) scattering process of the Au/Pt spherical particle fixing the Au core diameter (R = 30 nm black dots, R = 50 nm red dots, R = 70 nm blue dots) and increasing the Pt shell width d from 20 nm to 90 nm.

(7) AuPt: Figure 7a reports the picture of the structure of the Au/Pt core/shell spherical particle for which the simulations are performed with R the core radius and d shell width. The electromagnetic radiation of wavelength λ = 550 nm impinges on the particle from 0°. In addition, Figure 7b–m shows the calculated polar diagrams for the intensity of the scattered light from the Au/Pt core/shell spherical particle changing the Au core radius R and the Pt shell width (i.e., fixing R to 30 nm or 50 nm or 70 nm and increasing d from 20 nm to 90 nm). Finally, Figure 7n presents the calculated scattering efficiency values for the light (wavelength λ = 550 nm) scattering process of the Au/Pt spherical particle fixing the Au core diameter (R = 30 nm black dots, R = 50 nm red dots, R = 70 nm blue dots) and increasing the Pt shell width d from 20 nm to 90 nm.

In this case, we observe, in particular, that fixing R = 30 nm, the intensity I of the scattered radiation is maximum for θ = 0° when d = 20 nm, while I is maximum for θ = 180° when d = 50 nm, d = 70 nm, d = 90 nm. Fixing R = 50 nm, I is maximum for θ = 180° when d = 20 nm, d = 50 nm, d = 70 nm, and d = 90 nm, even if for d = 20 nm and d = 70 nm the intensity is only a little bit lower at θ = 0°. Fixing R = 70 nm, I is maximum for θ = 180° when d = 20 nm, d = 50 nm, and d = 70 nm, even if for d = 20 nm and d = 50 nm the intensity is only a little bit lower at θ = 0°. When R = 70 nm and d = 50 nm, I is the maximum for θ = 0°. The scattering efficiency, Q_scatt_, when fixed R = 30 nm, increases monotonically by increasing d from 20 nm to 90 nm; when fixed R = 50 nm, Q_scatt_ is about constant for d increasing from 20 nm to 50 nm and, then, monotonically increases by increasing d from 50 nm to 90 nm; when fixed R = 70 nm, Q_scatt_ decreases by increasing d from 20 to 50 nm and, then, monotonically increases by increasing d from 50 nm to 90 nm. The lowest scattering efficiency is obtained for R = 30 nm and d = 20 nm; the highest scattering efficiency is obtained for R = 50 nm and d = 90 nm; hence this is the best configuration for light-scattering. Moreover, the scattering efficiencies obtained for R = 30 nm and d = 90 nm and for R = 50 nm and d = 70 nm are about the same, corresponding to a total size of 120 nm.

**Figure 8:**
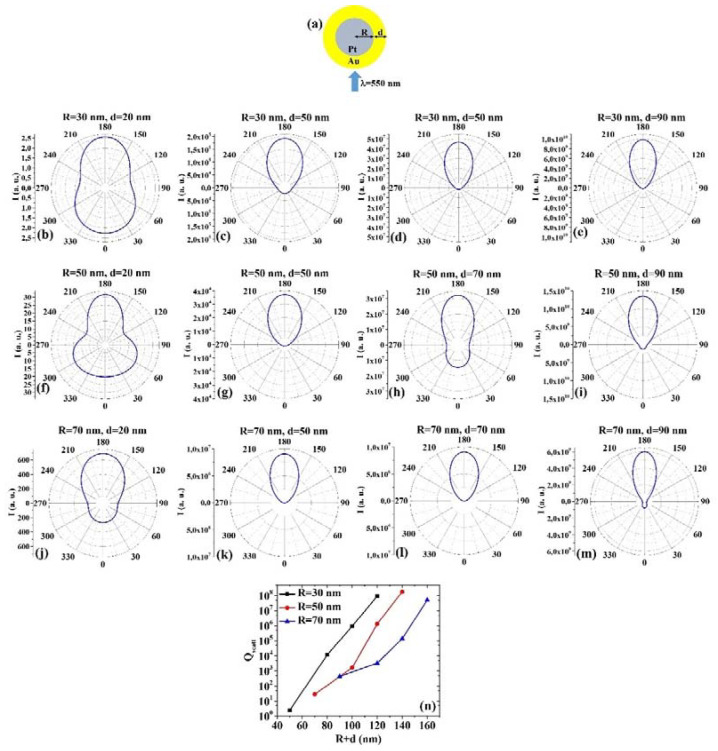
(**a**) Scheme of the structure of the simulated Pt/Au core/shell spherical particle with core radius R and shell width d and electromagnetic radiation of wavelength λ = 550 nm impinging on the particle from 0°. (**b**–**m**) Calculated polar diagrams for the intensity of the scattered light from the Pt/Au core/shell spherical particle changing the Pt core radius R and the Au shell width: (**b**–**e**) R = 30 nm and d = 20 nm (**b**), d = 50 nm (**c**), d = 70 nm (**d**), d = 90 nm (**e**); (**f**–**i**) R = 50 nm and d = 20 nm (**f**), d = 50 nm (**g**), d = 70 nm (**h**), d = 90 nm (**i**); (**j**–**m**) R = 70 nm and d = 20 nm (**j**), d = 50 nm (**k**), d = 70 nm (**l**), d = 90 nm (**m**). (**n**) Calculated scattering efficiency for the light (wavelength λ = 550 nm) scattering process of the Pt/Au spherical particle fixing the Pt core diameter (R = 30 nm black dots, R = 50 nm red dots, R = 70 nm blue dots) and increasing the Au shell width d from 20 nm to 90 nm.

(8) PtAu: Figure 8a reports the picture of the structure of the Pt/Au core/shell spherical particle for which the simulations are performed with R the core radius and d shell width. The electromagnetic radiation of wavelength λ = 550 nm impinges on the particle from 0°. In addition, Figure 8b–m shows the calculated polar diagrams for the intensity of the scattered light from the Pt/Au core/shell spherical particle changing the Pt core radius R and the Au shell width (i.e., fixing R to 30 nm or 50 nm or 70 nm and increasing d from 20 nm to 90 nm). Finally, Figure 8n presents the calculated scattering efficiency values for the light (wavelength λ = 550 nm) scattering process of the Pt/Au spherical particle fixing the Pt core diameter (R = 30 nm black dots, R = 50 nm red dots, R = 70 nm blue dots) and increasing the Au shell width d from 20 nm to 90 nm.

In this case, we observe, in particular, that fixing R = 30 nm, the intensity I of the scattered radiation is maximum for θ = 180° when d = 20 nm, d = 50 nm, d = 70 nm, d = 90 nm, however in the case d = 20 nm, the intensity at 0° is only a little bit lower than at 180°. Fixing R = 50 nm, I is maximum for θ = 180° when d = 20 nm, d = 50 nm, d = 70 nm, and d = 90 nm. For R = 50 nm and d = 20 nm, I is higher (even if not maximum) also for θ = 60° and θ = 300°. Fixing R = 70 nm, I is maximum for θ = 180° when d = 20 nm, d = 50 nm, d = 70 nm and d = 90 nm. The scattering efficiency, Q_scatt_, monotonically increases monotonically for each fixed R (30, 50, 70 nm) by increasing d from 20 nm to 90 nm. The lowest scattering efficiency is obtained for R = 30 nm and d = 20 nm; the highest scattering efficiency is obtained for R = 50 nm and d = 90 nm being, so this configuration the best for light-scattering-based applications.

Comparing, for example, Figure 7b and Figure 8b, we can observe that the Pt/Au particle is more effective in scattering the radiation at 180° than the Au/Pt particle. However, both particles scatter the radiation equally effectively at 0°; comparing Figure 7h and Figure 8h, we can observe that the Au/Pt particle is more effective in scattering the radiation at 0° than the Pt/Au particle; comparing Figure 7k and Figure 8k, we can observe that the Au/Pt particle is more effective in scattering the radiation at 0° than the Pt/Au particle, while both particles scatter about equally the radiation at 180°; comparing Figure 7m and Figure 8m, we can observe that the Au/Pt particle is more effective in scattering the radiation at 0° than the Pt/Au particle, while the Pt/Au particles are more effective in scattering the radiation at 180° than the Au/Pt particle. However, comparing the plots of the scattering efficiency, it appears that, generally, the Pt/Au geometry is more efficient in scattering light than the Au/Pt one.

**Figure 9:**
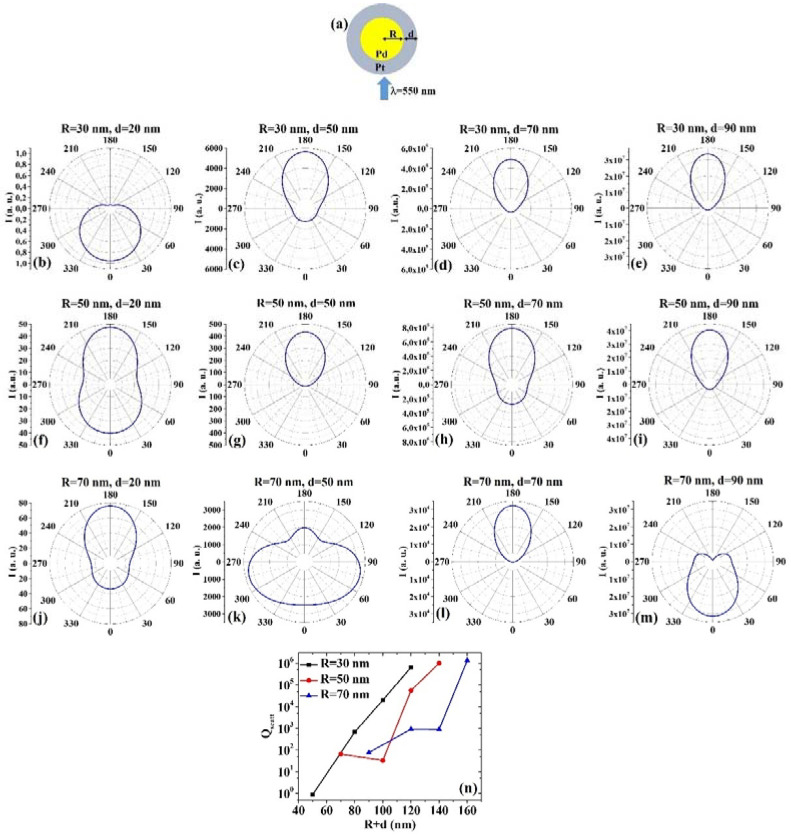
(**a**) Scheme of the structure of the simulated Pd/Pt core/shell spherical particle with core radius R and shell width d and electromagnetic radiation of wavelength λ = 550 nm impinging on the particle from 0°. (**b**–**m**) Calculated polar diagrams for the intensity of the scattered light from the Pd/Pt core/shell spherical particle changing the Pd core radius R and the Pt shell width: (**b**–**e**) R = 30 nm and d = 20 nm (**b**), d = 50 nm (**c**), d = 70 nm (**d**), d = 90 nm (**e**); (**f**–**i**) R = 50 nm and d = 20 nm (**f**), d = 50 nm (**g**), d = 70 nm (**h**), d = 90 nm (**i**); (**j**–**m**) R = 70 nm and d = 20 nm (**j**), d = 50 nm (**k**), d = 70 nm (**l**), d = 90 nm (**m**). (**n**) Calculated scattering efficiency for the light (wavelength λ = 550 nm) scattering process of the Pd/Pt spherical particle fixing the Pd core diameter (R = 30 nm black dots, R = 50 nm red dots, R = 70 nm blue dots) and increasing the Pt shell width d from 20 nm to 90 nm.

(9) PdPt: Figure 9a reports the picture of the structure of the Pd/Pt core/shell spherical particle for which the simulations are performed with R the core radius and d shell width. The electromagnetic radiation of wavelength λ = 550 nm impinges on the particle from 0°. In addition, Figure 9b–m shows the calculated polar diagrams for the intensity of the scattered light from the Pd/Pt core/shell spherical particle changing the Pd core radius R and the Pt shell width (i.e., fixing R to 30 nm or 50 nm or 70 nm and increasing d from 20 nm to 90 nm). Finally, Figure 9n presents the calculated scattering efficiency values for the light (wavelength λ = 550 nm) scattering process of the Pd/Pt spherical particle fixing the Pd core diameter (R = 30 nm black dots, R = 50 nm red dots, R = 70 nm blue dots) and increasing the Pt shell width d from 20 nm to 90 nm.

In this case, we observe, in particular, that fixing R = 30 nm, the intensity I of the scattered radiation is maximum for θ = 0° when d = 20 nm, while it is maximum for θ = 180° when d = 50 nm, d = 70 nm, d = 90 nm. Fixing R = 50 nm, I is maximum for θ = 180° when d = 20 nm, d = 50 nm, d = 70 nm, and d = 90 nm, however for d = 20 nm, the intensity is high also for θ = 0°. Fixing R = 70 nm, I is maximum for θ = 180° when d = 20 nm and d = 70 nm and d = 90 nm, while I is maximum for θ = 0° when d = 90 nm. In addition, when R = 70 nm and d = 50 nm, I is maximum for θ = 75° and θ = 285°. The scattering efficiency, Q_scatt_, monotonically increases when fixed R = 30 nm and increases d from 20 nm to 90 nm. Fixed R = 50 nm, Q_scatt_ decreases by increasing d from 20 nm to 50 nm and, then, monotonically increases by increasing d from 50 nm to 90 nm. Fixed R = 70 nm, Q_scatt_ increases by increasing d from 20 nm to 50 nm; then it is constant by increasing d from 50 nm to 70 nm, and, finally, it increases by increasing d from 70 nm to 90 nm. The lowest scattering efficiency is obtained for R = 30 nm and d = 20 nm; the highest scattering efficiency is obtained for R = 70 nm and d = 90 nm, even if the scattering efficiencies obtained for R = 30 nm and d = 90 nm, for R = 50 nm and d = 90 nm, for R = 70 nm and d = 90 nm are very similar, hence these geometries are equivalent in assuring the highest efficiency in light-scattering-based applications.

**Figure 10:**
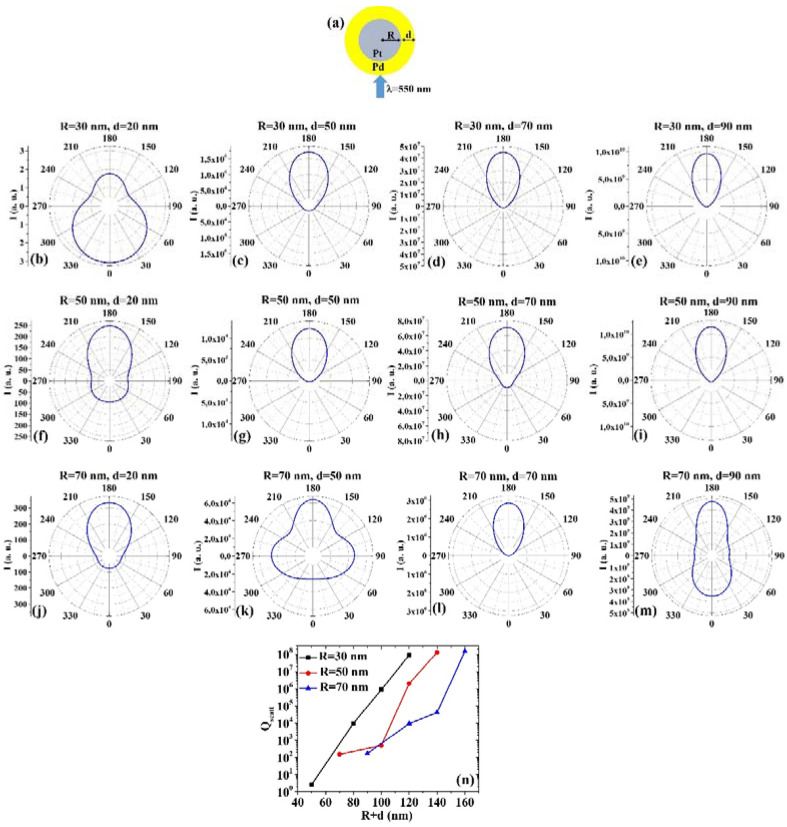
(**a**) Scheme of the structure of the simulated Pt/Pd core/shell spherical particle with core radius R and shell width d and electromagnetic radiation of wavelength λ = 550 nm impinging on the particle from 0°. (**b**–**m**) Calculated polar diagrams for the intensity of the scattered light from the Pt/Pd core/shell spherical particle changing the Pt core radius R and the Pd shell width: (**b**–**e**) R = 30 nm and d = 20 nm (**b**), d = 50 nm (**c**), d = 70 nm (**d**), d = 90 nm (**e**); (**f**–**i**) R = 50 nm and d = 20 nm (**f**), d = 50 nm (**g**), d = 70 nm (**h**), d = 90 nm (**i**); (**j**–**m**) R = 70 nm and d = 20 nm (**j**), d = 50 nm (**k**), d = 70 nm (**l**), d = 90 nm (**m**). (**n**) Calculated scattering efficiency for the light (wavelength λ = 550 nm) scattering process of the Pt/Pd spherical particle fixing the Pt core diameter (R = 30 nm black dots, R = 50 nm red dots, R = 70 nm blue dots) and increasing the Pd shell width d from 20 nm to 90 nm.

(10) PtPd: Figure 10a presents the picture of the structure of the Pt/Pd core/shell spherical particle for which the simulations are performed with R the core radius and d shell width. The electromagnetic radiation of wavelength λ = 550 nm impinges on the particle from 0°. In addition, Figure 10b–m shows the calculated polar diagrams for the intensity of the scattered light from the Pt/Pd core/shell spherical particle changing the Pt core radius R and the Pd shell width (i.e., fixing R to 30 nm or 50 nm or 70 nm and increasing d from 20 nm to 90 nm). Finally, Figure 10n presents the calculated scattering efficiency values for the light (wavelength λ = 550 nm) scattering process of the Pt/Pd spherical particle fixing the Pt core diameter (R = 30 nm black dots, R = 50 nm red dots, R = 70 nm blue dots) and increasing the Pd shell width d from 20 nm to 90 nm.

In this case, we observe, in particular, that fixing R = 30 nm, the intensity I of the scattered radiation is maximum for θ = 0° when d = 20 nm, while it is maximum for θ = 180° when d = 50 nm, d = 70 nm, d = 90 nm. Fixing R = 50 nm, I is maximum for θ = 180° when d = 20 nm, d = 50 nm, d = 70 nm, and d = 90 nm. Fixing R = 70 nm, I is maximum for θ = 180° when d = 20 nm, d = 50 nm, d = 70 nm and d = 90 nm. In addition, when R = 70 nm and d = 50 nm, I is high (even if not maximum) for θ = 90° and θ = 270°. When R = 70 nm and d = 50 nm, I is high (even if not maximum) for θ = 0°. The scattering efficiency, Q_scatt_, monotonically increases by increasing d when fixed R = 30 nm, R = 50 nm and R = 70 nm. The lowest scattering efficiency is obtained for R = 30 nm and d = 20 nm; the highest scattering efficiency is obtained for R = 70 nm and d = 90 nm, even if the scattering efficiencies obtained for R = 30 nm and d = 90 nm, for R = 50 nm and d = 90 nm, for R = 70 nm and d = 90 nm are very similar, hence these geometries are equivalent in assuring the highest efficiency in light-scattering-based applications.

Comparing, for example, Figure 9b and Figure 10b, we can observe that the Pt/Pd particle is more effective in scattering the radiation at 180° than the Pd/Pt particle; however, both particles scatter the radiation similarly at 0°; comparing Figure 9f and Figure 10f, we can observe that the Pd/Pt particle is more effective in scattering the radiation at 0° than the Pt/Pd particle; however, both particles scatter the radiation similarly at 180°; comparing Figure 9k and Figure 10k, we can observe that the Pt/Pd particle is more effective in scattering the radiation at 180° than the Pd/Pt particle; however the Pt/Pd particle reaches the maximum scattered intensity at 180° and the Pd/Pt particle at 75° and 285°; comparing Figure 9m and Figure 10m, we can observe that the Pd/Pt particle is more effective in scattering the radiation at 0° than the Pt/Pd particle, while the Pt/Pd particles are more effective in scattering the radiation at 180° than the Pd/Pt particle. However, comparing the plots of the scattering efficiency, it appears that, generally, the Pt/Pd geometry is more efficient in scattering light than the Pd/Pt one.

## 4. Conclusions

In this work, we reported theoretical results of angle-dependent light-scattering intensity and scattering efficiency for AuAg, CuAg, AuPd, AuPt, PdPt, AgAu, AgCu, PdAu, PtAu, PtPd spherical core–shell nanoparticles by changing the core and shell sizes. For various geometrical conditions, the I(θ) diagram and scattering efficiency Q_scatt_ were calculated. Combining the I(θ) and Q_scatt_ information, the best geometry for the bimetallic core–shell nanoparticles can be chosen for a specific application involving particular light-scattering properties of the system.

## Figures and Tables

**Table 1:** Values for the real part, n, and imaginary part, k, of the refractive index of the materials composing the core–shell particles (corresponding to the electromagnetic radiation wavelength λ = 550 nm) and used for the calculations [71].

λ = 550 nm	Au	Ag	Cu	Pd	Pt
n	0.42415	0.059582	1.0066	1.6412	0.46521
k	2.4721	3.5974	2.5823	3.8455	5.1073

## Data Availability

All the data are just available in the present article.
